# Spatial clustering in the spatio-temporal dynamics of endemic cholera

**DOI:** 10.1186/1471-2334-10-51

**Published:** 2010-03-06

**Authors:** Diego Ruiz-Moreno, Mercedes Pascual, Michael Emch, Mohammad Yunus

**Affiliations:** 1 Department of Ecology and Evolutionary Biology, University of Michigan, Ann Arbor, MI, USA; 2 Howard Hughes Medical Institute, 4000 Jones Bridge Road Chevy Chase, MD, USA; 3 Department of Geography, University of North Carolina, Chapel Hill, NC, USA; 4 ICDDR, B, Centre for Health and Population Research, Dhaka, Bangladesh

## Abstract

**Background:**

The spatio-temporal patterns of infectious diseases that are environmentally driven reflect the combined effects of transmission dynamics and environmental heterogeneity. They contain important information on different routes of transmission, including the role of environmental reservoirs. Consideration of the spatial component in infectious disease dynamics has led to insights on the propagation of fronts at the level of counties in rabies in the US, and the metapopulation behavior at the level of cities in childhood diseases such as measles in the UK, both at relatively coarse scales. As epidemiological data on individual infections become available, spatio-temporal patterns can be examined at higher resolutions.

**Methods:**

The extensive spatio-temporal data set for cholera in Matlab, Bangladesh, maps the individual location of cases from 1983 to 2003. This unique record allows us to examine the spatial structure of cholera outbreaks, to address the role of primary transmission, occurring from an aquatic reservoir to the human host, and that of secondary transmission, involving a feedback between current and past levels of infection. We use Ripley's K and L indices and bootstrapping methods to evaluate the occurrence of spatial clustering in the cases during outbreaks using different temporal windows. The spatial location of cases was also confronted against the spatial location of water sources.

**Results:**

Spatial clustering of cholera cases was detected at different temporal and spatial scales. Cases relative to water sources also exhibit spatial clustering.

**Conclusions:**

The clustering of cases supports an important role of secondary transmission in the dynamics of cholera epidemics in Matlab, Bangladesh. The spatial clustering of cases relative to water sources, and its timing, suggests an effective role of water reservoirs during the onset of cholera outbreaks. Once primary transmission has initiated an outbreak, secondary transmission takes over and plays a fundamental role in shaping the epidemics in this endemic area.

## Background

The spatial distribution of cases for infectious diseases that are environmentally driven, such as those of water-borne and vector-borne transmission, reflect the combined effect of environmental heterogeneity and disease dynamics. As such, spatial patterns can be used to examine hypotheses on routes of transmission at different scales [1-3]. For example, long-term disease data at the population level for multiple cities and towns, or presence-absence data in counties or districts, have provided numerous insights on patterns of propagation at the relatively large scales of countries and even transcontinental couplings [1,4-6]. Detailed spatial records at the individual level have a long tradition at the interface of geography and epidemiology, with one of the best known examples found in the discovery of cholera's water source by Snow [7]. However, these detailed data have been typically short-term and limited to particular epidemics. Longer-term data sets at the high-resolution of individuals but spanning multiple epidemics are becoming available, with the current interest in transmission networks and the interface of Geographic Information Systems and disease patterns. This type of data makes possible the description of how spatial patterns and clustering in particular, change in time, as a first step towards addressing the spatio-temporal dynamics of disease at a high resolution, as well as the coarse-graining of these dynamics, from small, individual, to large, population, levels.

In general, higher case numbers are expected in areas surrounding pathogen sources, and lower ones, in areas far away from these sources. For example, for vector-borne diseases, the clustering of cases might occur nearby the specific habitat of the vector, and for water-borne diseases, in the proximity of infected aquatic reservoirs. However, patterns are complicated by the fact that transmission can occur from both environmental reservoirs and previously infected individuals, reflecting the dynamics of transmission, human behavior and environmental variability. For cholera, an acute diarrheal infectious disease caused by the bacterium *Vibrio cholerae*, two routes of transmission have been proposed [8], the first one from aquatic reservoirs in the environment, and the second one, from previously infected individuals. To address the respective roles of these two routes of transmission, this paper examines the clustering patterns of cholera and how they vary in time with an extensive data set for a rural area of Bangladesh, covering twenty-one years and groups of households in the landscape.

Although the cholera bacterium has an enormous diversity with more than 200 serogroups classified based on the somatic O antigen, only two such groups, O1 and O139, are pathogenic [9-11]. *V. cholerae *O1 can be subdivided in turn into two biotypes (phenotypes), classical and El Tor [12]. The O1 serogroup can also be subdivided into serotypes (based on antigenic responses) called Ogawa, Inaba and the extremely rare Hikojima [10]. Moreover, the Bengal strain, *V. cholerae *O139, appeared in 1992 [13] presumably by horizontal gene transfer between O1 and another O serogroup [10]. Nowadays, evidence from field studies and epidemiological models, supports the fact that the classical variant is more infective than the El Tor variant. However, the El Tor variant is more resilient outside the human host, surviving in the environment for a longer period than the classical strain [14,15]. Recent evidence suggests that *V. cholerae *O139, which exhibits no cross immunity with the O1 serogroups, performs better in both, the environment and the human gastrointestinal tract [16], raising questions on its short epidemic period.

Survivorship of the pathogens, availability of susceptible individuals and temporal immunity appear to be important processes shaping the pronounced seasonality of cholera. In non-endemic locations (like Peru, Brazil and several countries in Africa), epidemics are confined to the warm and rainy season. By contrast, in endemic areas in Bangladesh and former Bengal, two epidemic peaks occur each year corresponding to the warm seasons before and after the monsoonal rains [11,17]. A full explanation of this complex seasonal pattern is still lacking, and a number of environmental and ecological drivers have been proposed (for example, the presence of environmental cholera phages, the degree of crowdedness and availability of susceptible individuals) [18]. For estuarine regions of Bangladesh with two peaks per year, evidence supports a relationship between the initiation of the spring peak of cholera and favorable environmental conditions for the bacterial population, such as warmer temperatures [19]. A dilution effect would then drive the reduction of cholera during the monsoonal season [17], although recent mathematical models of historical mortality time series, suggest an alternative explanation in which susceptibles are depleted by a large number of asymptomatics and short-term immunity [20]. Finally, an increase in human-to-human contact, as a consequence of the post monsoonal floods might be an important factor in the second epidemic peak [21].

Infectivity and survivorship affects the spatial distribution of *V. cholerae *and its availability to colonize susceptible individuals. From this perspective, an initial study on the spatial occurrence of cholera cases by Glass and colleagues [22] provided a detailed description of cholera epidemics during the period from 1966 to 1980. This study identified villages with high and low cholera risk. Based on these findings, Miller and colleagues proposed a cholera model with two routes of transmission, primary and secondary, respectively [8]. Primary transmission occurs as the result of eating contaminated shellfish or aquatic plants, and drinking contaminated water. Secondary transmission results instead from a 'person-to-person' infection. Thus, primary transmitted cases should be scattered in space, occurring almost simultaneously in different areas with no apparent interconnection, and should be located relatively close to water sources. These initial cases would be followed by the clustering of secondary transmitted cases [8]. Miller and colleagues further proposed that primary transmission shapes the seasonal patterns in endemic areas, whereas secondary transmission determines the level of infection.

It is important to note that secondary transmission can occur directly or indirectly (i.e., via fecally contaminated water), and that it can involve the same aquatic reservoirs than those of primary transmission. These two modes of transmission effectively represent the two extremes of a possible continuum, with the key distinction being the degree of memory in the current force of infection of previous levels of infection. Only the secondary route possesses this memory, and this is the sense in which 'human-to-human' transmission should be interpreted.

The study of spatial cholera patterns by Craig further addressed the existence of clustering with case data from 1970 to 1982 at the level of *baris*, patrilineally related household groups [23]. A significant excess of cases was detected at short distances, "within *baris*" of less than 250 meters, and short time intervals (3 and 10 days). However, this analysis did not take into account that the spatial location of cases is not random and that the distribution of the *baris *themselves must be taken into account. It also did not consider the timing between the occurrence of clusters and the epidemic peaks, and the nature of the clustering (positive or negative). Nevertheless, Craig's study was the first description of cholera's spatial distribution in an endemic area at high resolution.

We further examine here the spatio-temporal distribution of cholera cases in Matlab, Bangladesh, to address the occurrence of clustering during epidemic years. We specifically consider the relationships between cluster size and epidemic intensity, between the timing of clustering and the epidemic curve, and that between clusters of cases and water sources. Primary transmission from water reservoirs is believed to occur when environmental conditions are favorable [19,24]. A spatial association between cholera cases and water sources should be observed if primary transmission drives the development of epidemics, with no significant spatial correlation between observed cholera cases at the beginning of the outbreak [8]. Because water sources (rivers, canals and water ponds of diverse size) are found almost everywhere and are extremely abundant in our study area, they are not likely to impose significant spatial constraints on the occurrence of cases. Thus, cases generated by environmental transmission should occur almost anywhere. By contrast, once epidemics have reached a threshold size, secondary transmission should dominate the dynamics, generating spatial associations between cases. This pattern should be most pronounced during the fall peak after the monsoonal rains, when secondary transmission has been suggested to play a more important role [17,21], since flooding disrupts sanitary conditions and concentrates human populations in the landscape. This hypothesis would predict a significant clustering of cases during the fall peak and a weak spatial association between cholera cases and water sources. We examine these predictions here.

## Methods

**Figure 1:**
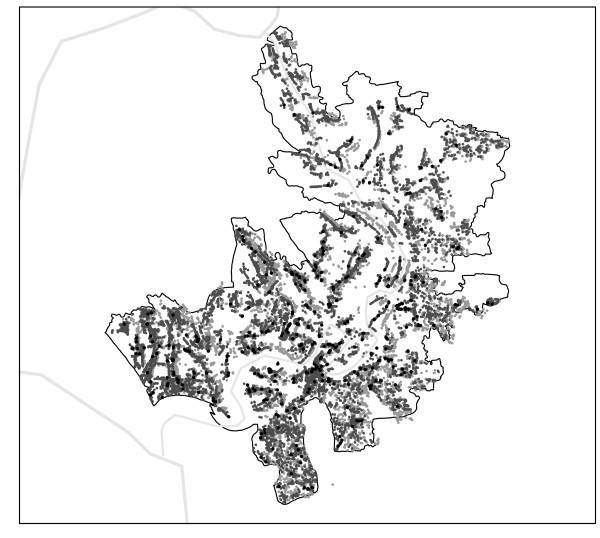
**Study Area**. The Matlab rural area. Light gray points are mapped water sources (ponds), dark gray points are *baris *and, as example, black points map cases from one epidemic of cholera classical Ogawa. Black lines represent the limits of the study area and light gray lines are rivers.

The data for this study come from the rural area of Bangladesh known as Matlab, located about 50 km south-east of Dhaka, the capital of the country. The study area is adjacent to the confluence of the Meghna and the Ganges Rivers, and is bisected by the Dhonagoda River into two approximately equal parts. Numerous canals and water ponds are present in the whole region (Fig. 1).

People in this area live in groups of patrilineally related households known as *bari*, with an average of six households [25]. Starting in 1994, a spatial database was created to facilitate the analysis of health and population research. All the *baris *were identified by a census number by the ICDDR, B (International Centre for Diarrhoeal Disease Research, Bangladesh) demographic surveillance system, allowing incidence data to be linked to the specific location of a single *bari *[25,26]. Complementing the information on *baris*, geographic coordinates of major water sources (i.e., big water ponds) were also acquired. Untreated surface water from ponds and rivers is used by villagers for household uses, but there is conflicting evidence about its use for drinking purposes [26,27], given that water from tube wells has been found to be contaminated with arsenic. Our analyses focus on the cholera cases from 1983 to 2004 for 8340 *baris *of this spatial database.

Health data were obtained from both surveillance and laboratory analyses. Individual health and demographic surveillance data were regularly collected for all individuals living in the Matlab study area. The Matlab hospital is the only diarrheal treatment center in the rural area, providing free treatment to all patients. Stool samples for all patients were collected and screened for enteric pathogens in the laboratory. Cholera cases were registered using the date of admission to the hospital and both biotype and serotype were reported. Thus, with approval of the ICDDR, B for this study we only used anonymous surveillance data (in agreement with the World Medical Association Medical Ethics Manual).

**Figure 2:**
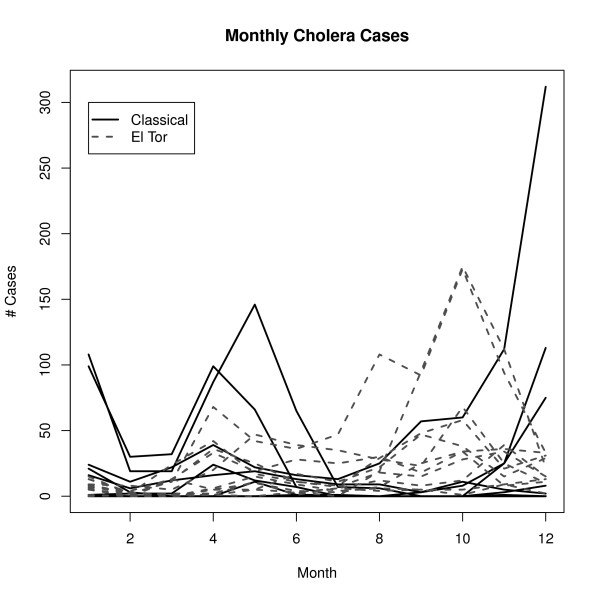
**Temporal Dynamics of Cholera epidemics**. The different lines represent annual cholera epidemics, solid black lines correspond to epidemics of classical (Inaba and Ogawa) strain, dashed dark gray lines show El Tor epidemics (Inaba and Ogawa). Variability is strongly marked, not only inter-annual or seasonality, but also intra-annual variability in both the timing of the outbursts and magnitude of the epidemics.

During the study period, 7241 cholera cases were recorded (1972 cases correspond to classical O1 and 5269 cases, to El Tor O1). Temporal variability is evident at both seasonal and inter-annual scales in the data (Fig. 2). The spatio-temporal information for all cases was used to analyze the spatial clustering of cholera cases *per se *(clustering of cases from now on), the spatial clustering of cholera cases with respect to the available water sources locations (clustering of cases-water from now on) and the timing between the occurrence of significant spatial associations and the dynamics of the epidemics.

Initially, we studied the spatial distribution of cholera cases in order to detect the presence of spatial trends (i.e., first order trends, see Additional File 1). Although the distribution of cholera cases seems to display spatial trends (Additional File 2, Fig. S1), when this distribution is corrected by the spatial heterogeneity of the *baris*, such spatial trends are not statistically significant (Additional File 2, Fig. S2-S9). Some spatial trends have been previously reported [28] but, different spatial and temporal scales of aggregation for the data were considered in those studies. In addition, by performing Monte Carlo replications to evaluate the significance of the results (see details bellow), we explicitly considered the heterogeneity in the spatial distribution of the *baris*, an underlying pattern that was not taken into account in previous analyses.

The clustering of cases was studied using spatial statistics derived from the popular Ripley's K index [3,29]. The Ripley's K index quantifies nonrandom clustering patterns (by estimating the second order effects from an observed point pattern). For a particular region ℜ with area *R*, where *n *events (i.e., infections) have been observed, the Ripley's K index can be estimated as:(1)

where the function *I*_*h*_(*d*_*ij*_) indicates whether or not the events *i *and *j *occurred within a distance less than or equal to *h*; and *w*_*ij *_is an edge correction to handle arbitrary shaped regions [29,30]. It is important to notice that the K function considers the intensity, the mean number of events (infections) per unit area, of the process under analysis, and therefore the detection of a clustered distribution means that the number of cases (more properly, events) is above the expected density of cases at that particular scale.

The K function can be easily transformed into the well-known L function [29], estimated by:(2)

When (*h*) is plotted against *h*, the peaks for positive values indicate spatial attraction of events, or clustering, whereas the troughs for negative values indicate the spatial repulsion, or regularity, at the corresponding scale of distance *h *in each case. Zero values indicate a random spatial distribution. The Ripley's K function provided in the R package "spatstat" was used for these analyses [31]. Ripley's K and L functions were calculated by aggregating the daily cholera data into temporal windows ranging from 2 to 17 days. This temporal window represented potential infectious periods [12]. In addition this also allowed the inclusion of sufficient cases to statistically evaluate clustering of cases for the different strains.

**Figure 3:**
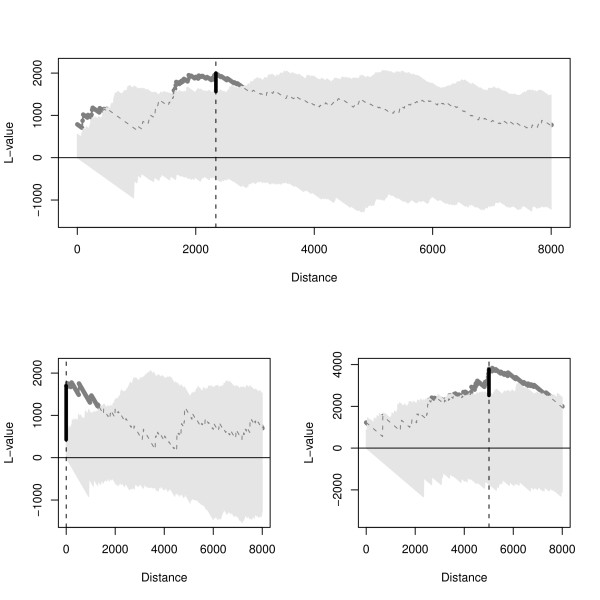
**Ripley's L function for an particular epidemic**. These three examples show variability and characteristics of the spatial clustering. Plotted in dark gray, the Ripley's L function for the observed data against distance. The Ripley's L function for observed data (dark gray) is plotted bold when statistically significant (i.e., outside the bootstrapping envelop), but otherwise is dashed. The light gray area was defined by the 10000 bootstrapping replications, these replications were constructed using all the cases for a particular epidemic. The most significant cluster size, or simply cluster size, is defined as the distance at which the difference between the bootstrapping envelope and the observation is maximum (black bold vertical segment), this is indicated by the vertical dashed line in the figure. The top panel shows a case where the cluster size is relatively large (2340 meters) but small scale significant clusters are also present. The bottom-left shows a cluster size with small scale (50 meters) without the occurrence of clusters at large scales. The bottom-right displays a large cluster size (5010 meters) without clusters at small scales.

Two important factors must be considered in the spatial analysis of cholera cases. First, their spatial location is constrained by the spatial location of *baris*. Second, the *baris *themselves are not homogeneously distributed in the Matlab region. One way to take into account these deviations from the original tests, is to perform Monte Carlo replications for the sequence of cholera cases over time, and thus evaluate significance accounting for the underlying landscape. Spatial clustering and repulsion are statistically significant when the observed L values are outside the envelope defined by the maxima and minima of the Monte Carlo replications (technical details in Fig. 3). The spatial distances for which the Ripley's functions were calculated (the maximum value for *h *in formulas (1) and (2)) were restricted to below 8 kilometers because of the size of the study area (which spans approximately 18 kilometers in longitude and 22 kilometers in latitude).

The spatial clustering and/or repulsion of cases-water was studied by using a cross K function. For this analysis, cholera cases and water sources are considered as two different types of events. Rivers and canals were not included in this analysis. Distance to main rivers have been proven to be unrelated with cholera cases in this area [26]. Frequent dryness of canals make them a very unlikely source of disease.

Under the assumption of independence between types of events (cholera cases and water sources), the location of one type should be random with respect to that of the other type, regardless of the overall spatial distribution of either type. Hence if cases originated from water sources, the location of cases will be correlated with the location of water sources, and therefore the K function will identify such relationship as clustering at particular scales. Following the previous reasoning, the L function can be used to consider the interaction of two (or more) types of events, to evaluate spatial attraction, independence or repulsion (see [29] chapter 4 for details). An equivalent bootstrapping technique was used to evaluate the statistical significance of the clustering of cases-water.

For both spatial analyses (clustering of cases and cases-water), the characteristic cluster size was defined as the distance corresponding to the largest significant difference (i.e., the most significant difference). Hence, the largest difference between the observed L values and the corresponding superior envelope from the bootstrapping was considered as the characteristic cluster size [29]. Note that during the course of an outbreak, significant clusters may occur at several scales (Fig. 3), but only the most significant is considered for the analyses presented in this work.

During the study period, several cholera outbreaks with different intensity occurred. To reduce the variability presented in the data we defined (and analyzed) epidemics as outbreaks that surpassed 30 new cases during the course of a week. This is based on the observed distribution of epidemic sizes. Although epidemics exhibited the typical seasonal pattern of two peaks per year during most of the study period in this endemic region, the timing of initiation of these peaks exhibited variability from year to year. Based on the ideas from Cazelles and Stone [32], we rescaled time for each epidemic as follows: Each epidemic was mapped onto the interval |0, 4π|, with the beginning mapped to time 0, the first (fall) peak to π, the inter-peak decay to 2π, the second (spring) peak to 3π and finally the end of the epidemic to 4π. Four stages (1 to 4) were thus defined, with stages 1 and 3 corresponding to the rise of epidemics (fall and spring), and stages 2 and 4, to their decay. Given that no biological cross-immunity between O1 and O139 strains has been reported [33], the dynamics of O139 epidemics can be considered independent from that of 01 and was therefore not considered here.

## Results

**Figure 4:**
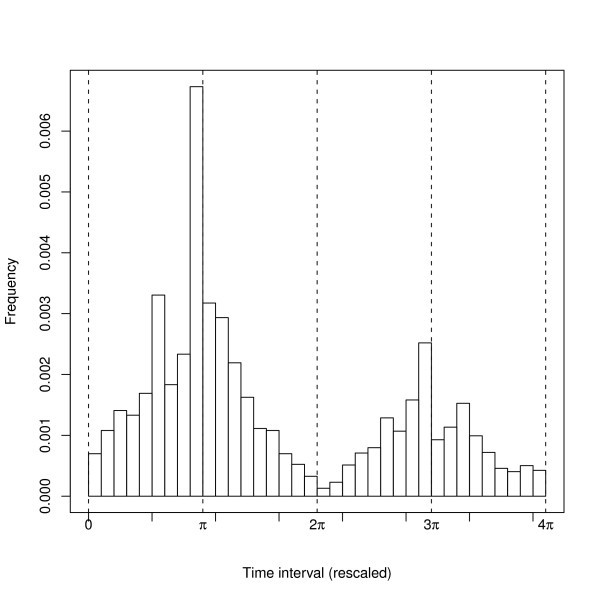
**Cholera seasonality**. Seasonal variation is shown on the mean number of cholera cases (El Tor Inaba) during the epidemics. In this plot the time has been rescaled and hence outbreaks from several years were aggregated. The vertical lines show beginning, first peak, the inter epidemic trough, second peak and end of the average occurrence of cases.

During the period of study, cholera epidemics in Matlab exhibited the typical pattern of two peaks per year (Fig. 4) reported for other endemic areas in Bangladesh [17] and some areas in historical Madras [21]. This pattern was observed regardless of the specific strain (Fig. 2). Moreover, the fall peak (corresponding to π in the figures) is bigger than the spring peak (in Fig. 4, Kruskal-Wallis test χ ^2 ^= 21.5, *df = *1, *p*-*value = *3e-06, for a temporal aggregation of 7 days, see Additional File 1, Table S2 for other examples). The decay of cholera cases during the monsoon season usually leads to a fade-out of the epidemic, while the decrease in the winter rarely reaches extinction.

**Figure 5:**
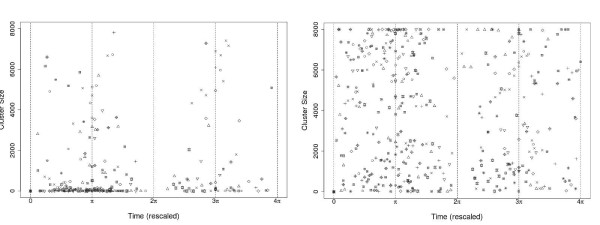
**Cluster size for the different epidemics**. Time has been rescaled and π represents the Fall peak and 3π the Spring peak. Cluster Size for cases is shown in (a) and cluster size for cases-water in (b) Different symbols represent different epidemics a temporal window of 5 days for the disease data was used for this figure, but see additional figures for a disaggregation for the different epidemics and temporal windows. In (a) the occurrence of small clusters is clearly abundant, moreover medium size clusters (less than 6 Km) are less abundant and bigger clusters are rare, this pattern is not clear for (b). In addition, clusters clearly occur more often in the Fall peak and in the Spring.

We present representative results based on the (most significant) cluster size for cholera cases (see Methods), however it must be noticed that high variability is present in both data (Fig. 2) and results (Fig. 5). Given the small outbreaks of El Tor Ogawa variant, the results corresponding to this strain are not included here. All yearly epidemics display several significant clusters of cases with varying sizes. Moreover, cluster sizes fall into two dominant scales, a small spatial scale (hundreds of meters) and a relatively large scale (ranging from 3 to 6 Kilometers), bigger clusters are rare (Fig. 5a). These spatial scales are present independently from the temporal scale of aggregation of the cases (see Additional File 3, Fig. S10 and Additional File 4, Fig. S11, or Additional File 5 and Additional File 6 for high resolution version of those figures). In addition, significant clusters are not present in a continuous way (i.e., a random pattern is detected at several intermediate scales, Fig. 3 and Fig. 5a). Finally, the most significant cluster size exhibits high variability during the course of epidemics, but big cluster sizes correspond to peaks in epidemic size.

A less clear pattern is obtained for the clusters sizes corresponding to cases-water (Fig. 5b). Clusters of different sizes are present at different times, although few clusters seem to be present during the winter (corresponding to 2π in Fig. 5b).

**Figure 6:**
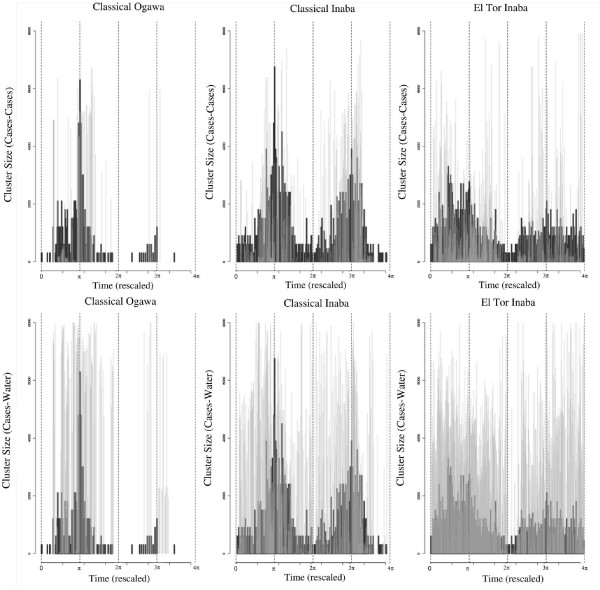
**Temporal dynamics of clustering and epidemic size**. Temporal dynamics of clustering and epidemic size averaged for all epidemics. Top row shows cluster size for cases in gray and epidemic size in black. Bottom row shows cluster size for cases-water in gray over epidemic size in black. Left column displays classical Ogawa data, center is classical Inaba and right column shows El Tor Inaba data.

Results for the dynamics of clustering are summarized in Fig. 6 where the dynamics of epidemic size and cluster size (for cases and cases-water) averaged over all epidemics for each particular strain are shown. Not surprisingly cluster size tracks epidemic size. Sudden changes in epidemic size are reflected in either cluster size of cases and/or cases-water sources. A closer look reveals some additional features. The size of the clustering of cases-water sources is, in general, bigger than that corresponding to clustering of cases. As expected significant clustering appears with some delay with respect to the onset of the epidemics (for both peaks) (see also Fig. 5). Clusters occur more frequently during the decay than during the onset of the epidemics (regardless of the peak), and also are more frequent during the fall than during the spring peak (regardless of the rate of increase of epidemics) (see Tables 1 and 2).

**Table 1:** Presence of significant clustering for cases.

		Proportion of time with sig. clustering of cases		
		
	Interval	classical Ogawa	classical Inaba	El Tor Inaba
Fall Peak	[0-π]	0.7278	0.9218	0.9722
	
	[π-2π]	0.8218	0.8889	0.9611

Spring Peak	[2π-3π]	0.6259	0.9278	0.8833
	
	[3π-4π]	0.7206	0.8111	0.9700

Proportion of time that significant clustering was exhibited (corrected for the presence of the disease) for clusters between cases.

**Table 2:** Presence of significant clustering for cases-water.

		Proportion of time with sig. clustering of cases-water		
		
	Interval	classical Ogawa	classical Inaba	El Tor Inaba
Fall Peak	[0-π]	0.8000	0.9665	0.9994
	
	[π-2π]	0.8851	0.9991	0.9996

Spring Peak	[2π-3π]	0.7143	0.9833	0.9667
	
	[3π-4π]	0.8676	0.8667	0.9992

Analogous to Table 1, but considering the clustering of cases with respect to water sources.

**Figure 7:**
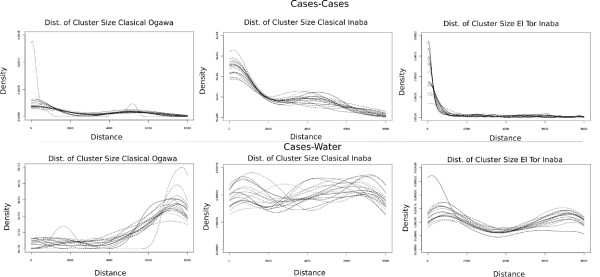
**Distribution of cluster sizes for different strains**. Top row shows cluster size for cases. Bottom row shows cluster size for cases-water. Left column displays classical Ogawa data, center is classical Inaba and right column shows El Tor Inaba data. Different lines represent different temporal aggregation of the data.

The distribution of cluster sizes exhibited by the different strains (over all epidemics) is shown in Fig. 7. The first row show the distribution of cluster sizes for cases and the second row, for cases-water. For classical Ogawa epidemics (Fig. 7 top-left) the distribution of cluster sizes for cases displays two characteristic sizes (around 200 meters and 5000 meters). The distribution of cluster sizes for cases-water on the other hand shows one typical scale around 7100 meters (Fig. 7 bottom-left). For classical Inaba epidemics (Fig. 7 top-center) the distribution of cluster sizes for cases displays also two characteristic sizes (around 200 meters and 4000 meters), but the distribution of cluster sizes for cases-water does not exhibit a characteristic scale (Fig. 7 bottom-center). The distribution of cluster sizes for cases of El Tor Inaba (Fig. 7 top-right) shows a small characteristic scale (150 meters), however two characteristic scales are found in the distribution of cluster sizes for cases-water (around 800 meters and 7000 meters).

## Discussion and Conclusions

Our results indicate that primary transmission sparks the start of epidemics at several distant locations more or less synchronously. This generates the clustering of cases only at big scales with respect to water sources, as opposed to small clusters close to a few punctual sources. Secondary transmission follows, and the clustering of cases at relatively small scales becomes dominant. At the larger scales, however, these new cases create a relatively homogeneous spatial distribution because of the multiple origins of seasonal outbreaks, and therefore clustering with respect to water sources persists for those scales most of the time. The onset of epidemics is therefore controlled by both secondary and primary transmission. However, rapid increases in the number of cases are clearly tracked by spikes in clustering, indicating that secondary transmission plays a fundamental role in the development of epidemics. These findings are consistent with the interpretation of cholera patterns previously proposed by Miller and colleagues [8].

Additional support for the fundamental role of secondary transmission during the onset of the epidemics is provided by the fact that the "epicenter" of the epidemic (calculated as the center of mass for the location of cases) moves during the course of the outbreaks (results not shown). A more constant epicenter would reflect the spread of epidemics from an initial source of contamination.

Spatial clustering practically disappears during inter-epidemic periods, as expected, with a few randomly spatially distributed cases (if any) occurring until the beginning of another outbreak (Fig. 5 and Fig. 6). The spatial clustering of cases is detected at several scales, with two dominant sizes, given by hundreds of meters and a few kilometers, consistent across the different epidemics. These two scales suggest the occurrence of long distance transmission events that might be triggered (among other causes) by the movement of asymptomatic individuals, given their importance during epidemics [20].

The frequency of cluster occurrence (Tables 1 and 2) showed that the clustering of cases-water occurs more often than that of cases. This difference is consistent with the observation of cases-water clustering from the very beginning of outbreaks as primary transmission triggers epidemics. It is also consistent with the clustering of cases emerging with some delay but still in the early stages of epidemics, as secondary transmission becomes established and dominates the dynamics (Fig. 5 and Fig. 6). This pattern exhibits small differences among the different strains. The contamination by secondary cases of new water sources could explain the persistence of clustering at large scales for cases-water.

The spatial clustering pattern observed during the decay of the outbreaks is sometimes indistinguishable from a random spatial pattern (Fig. 5 and Fig. 6). This could be due to several factors. One possibility is that, the increase in rainfall and associated dilution effect, might eliminate the spatial structure. In addition, heterogeneity in the recovery time of infected individuals and behavioral changes, induced by the presence of cases, would alter the observed spatial pattern. This random pattern could also reflect a failure of the chain of transmission of the disease, with the re-initiation of epidemics from environmental sources.

The lack of strong seasonality on the presence of cases-water clustering (Table 2) could reflect the effectiveness of water sources as a reservoir and their role as a 'link' between the clusters of secondary cases. Some of the variability in the effectiveness of water sources might result from strong El Niño-Southern Oscillation (ENSO) events which have been shown to be strongly associated with cholera epidemics [34].

This work presents results on the spatial distribution of cholera cases during twenty-one years in Matlab, Bangladesh, that support the role of secondary transmission in the dynamics of the disease. During the study period a mass vaccination program took place in Matlab [35-37]; the cholera vaccine appears to provide temporal immunity and reports suggest that herd immunity was present at some spatial scales [38-40]. Our analyses of spatial trends shows, however, that for the scales considered in this study the effects of immunization were not important (see supplementary material). The spatial information from this vaccination program, together with more detailed local information (i.e., sanitary conditions, education and socioeconomic status at the level of *baris *or even individuals), could be incorporated in further studies that address the localization of the clusters and disease risk. In particular, local clustering methods may be useful for analyzing the effects introduced by the construction of a flood control embankment that protects the northern half of the study area in the late1980's [41,42].

Finally, the analyses presented in this work are based on a static framework, where clustering is calculated from snapshots. A natural next step is to incorporate dynamical feedbacks between disease processes and the state of the population together with seasonality. Stochastic spatio-temporal models, similar to the ones developed for the advance of the "epidemic front" for rabies in the USA [2,43], are under construction. The rabies model identified in particular the consequences of spatial heterogeneity for the spread of the front of infection. Endemic dynamics such as those of cholera in Bangladesh require the development of stochastic models that not only describe the propagation of a front but are also able to include the "back-propagation" to areas with both previously uninfected (still susceptible) and/or recovered individuals. Nested models can be formulated to evaluate the role of primary and secondary transmission, as well as the importance of immunity and cross-immunity in determining the complex spatial patterns observed in cholera dynamics [44,45]. This work would extend the current scope of metapopulation and individual-based models of infectious diseases, to consider the stochastic dynamics that arise at high spatial resolution for diseases with temporary immunity. Such high resolution models would provide a basis to examine how to scale the dynamics up to the population level, to the simpler temporal models that are currently used for cholera and other infectious diseases in confronting time series data (e.g. [1,2,5,20,46-53]). These approaches will lead to a better understanding of the importance of spatial structure in the temporal dynamics of the disease.

## Competing interests

The authors declare that they have no competing interests.

## Authors' contributions

DRM designed the study. DRM and MP analyzed the data and drafted the manuscript. ME and MY collected the data. All authors provided critical comments for revision and the final version of the manuscript. All authors read and approved the final manuscript.

## Pre-publication history

The pre-publication history for this paper can be accessed here:

http://www.biomedcentral.com/1471-2334/10/51/prepub

## Supplementary Material

Additional file 1 Description of first order spatial analyses, including population size and number of cases and tables S1 and S2. Click here for file

Additional file 2 Figures S1, S2, S3, S4, S5, S6, S7, S8 and S9. Click here for file

Additional file 3 Additional Figure S10, showing clustering size between cases. Click here for file

Additional file 4 Additional Figure S11, showing clustering size cases-water. Click here for file

Additional file 5 High definition version of the addition Figure S11. Click here for file

Additional file 6 High definition version of the addition Figure S10. Click here for file
