# Supplementary material for: Spatial clustering in the spatio-temporal dynamics of endemic cholera
Source: BMC Infect Dis. 2010 Mar 6;10:51. doi: 10.1186/1471-2334-10-51 (PMC2846945; doi:10.1186/1471-2334-10-51)
Supplement: Additional file 1 — Description of first order spatial analyses, including population size and number of cases and tables S1 and S2. [file 1471-2334-10-51-S1.PDF]

Supplementary Material

Analysis of First Order trends in the dataset.

In order to study the presence of spatial trends in the data, spatial density of the data was calculated and the presence of trends was analyzed in all X or East-West, Y or North-South and XY or NW-SE or NE-SW directions. Estimates of densities, obtained by linear bi-dimensional interpolation, are shown in Figure S1. The spatial trends observed in Figure S1 could be due to heterogeneity on the spatial distribution of baris, therefore a Monte Carlo approach was used to determine significance. 1000 Monte Carlo replications were performed to evaluate the significance of the observed spatial trends. In each of these replications, disease cases were randomly assigned to spatial locations. The observed trends for the different directions (X, Y and XY) are shown in bold vertical lines in Figures S2, S3, S4 and S5 for Classical Inaba, Calssical Ogawa, El Tor Inaba and El Tor Ogawa respectively. All these observed values, fall inside the 5th-95th percentile intervals generated by the Monte Carlo distributions, and therefore are not statistically significant.

Spatial trends considering the distance to the Hospital were also analyzed, and again evaluated using 1000 Monte Carlo replications for significance. Significant trends were obtained for all the strains, however the magnitude of the trend made a difference of less than 1 reported cases (in excess or deficit) inside the study area. The importance of these trends result negligible for the clustering analysis (Table S1).

Table S1: Linear trend for reported cases as a function of distance to the hospital.

Strain	Fitted Linear Model $Cases = intercept + X * distance$		Maximum difference in number of cases due to this trend using the maximum distance (22006.22m)
	Intercept	X	
Classical Inaba	0.90000	-4.82E-005	-0.16
Classical Ogawa	0.90161	-4.62E-005	-0.16
El Tor Inaba	0.85500	-5.62E-005	0.00
El Tor Ogawa	0.70909	-3.62E-005	0.00

Relationship between population size and the reported number of cases.

The number of cases reported in baris that have more inhabitant does not show a clear relationship. Figures S6, S7, S8 and S9 show this relationship for Classical Inaba, Classical Ogawa, El Tor Inaba and El Tor Ogawa respectively.

Table S2: Results for Kruskal-Wallis test for different time aggregation of the data, showing the difference between the distribution of cases during the fall $[0-2\pi]$ and in the spring $[2\pi-4\pi]$.

Temporal Windows Size (days)	χ^2	df	p-value
2	19.1725	1	1.19E-05
3	20.6864	1	5.41e-06
4	17.1092	1	3.52E-05
5	22.4301	1	2.18E-06
6	75.3018	1	< 2.2e-16
7	21.5255	1	3.49E-06
8	20.5482	1	5.82E-06
9	20.0848	1	7.41E-06
10	27.7922	1	1.35E-07
11	53.9584	1	2.05E-13
12	16.9417	1	3.86E-05
13	20.8998	1	4.84E-06
14	50.5590	1	1.16E-12
15	88.0762	1	< 2.2e-16
16	76.3242	1	< 2.2e-16
17	105.8511	1	< 2.2e-16

Table S2
