# Supplementary material for: Spatial clustering in the spatio-temporal dynamics of endemic cholera
Source: BMC Infect Dis. 2010 Mar 6;10:51. doi: 10.1186/1471-2334-10-51 (PMC2846945; doi:10.1186/1471-2334-10-51)
Supplement: Additional file 2 — Figures S1, S2, S3, S4, S5, S6, S7, S8 and S9. [file 1471-2334-10-51-S2.PDF]

Supplementary Material: Figures

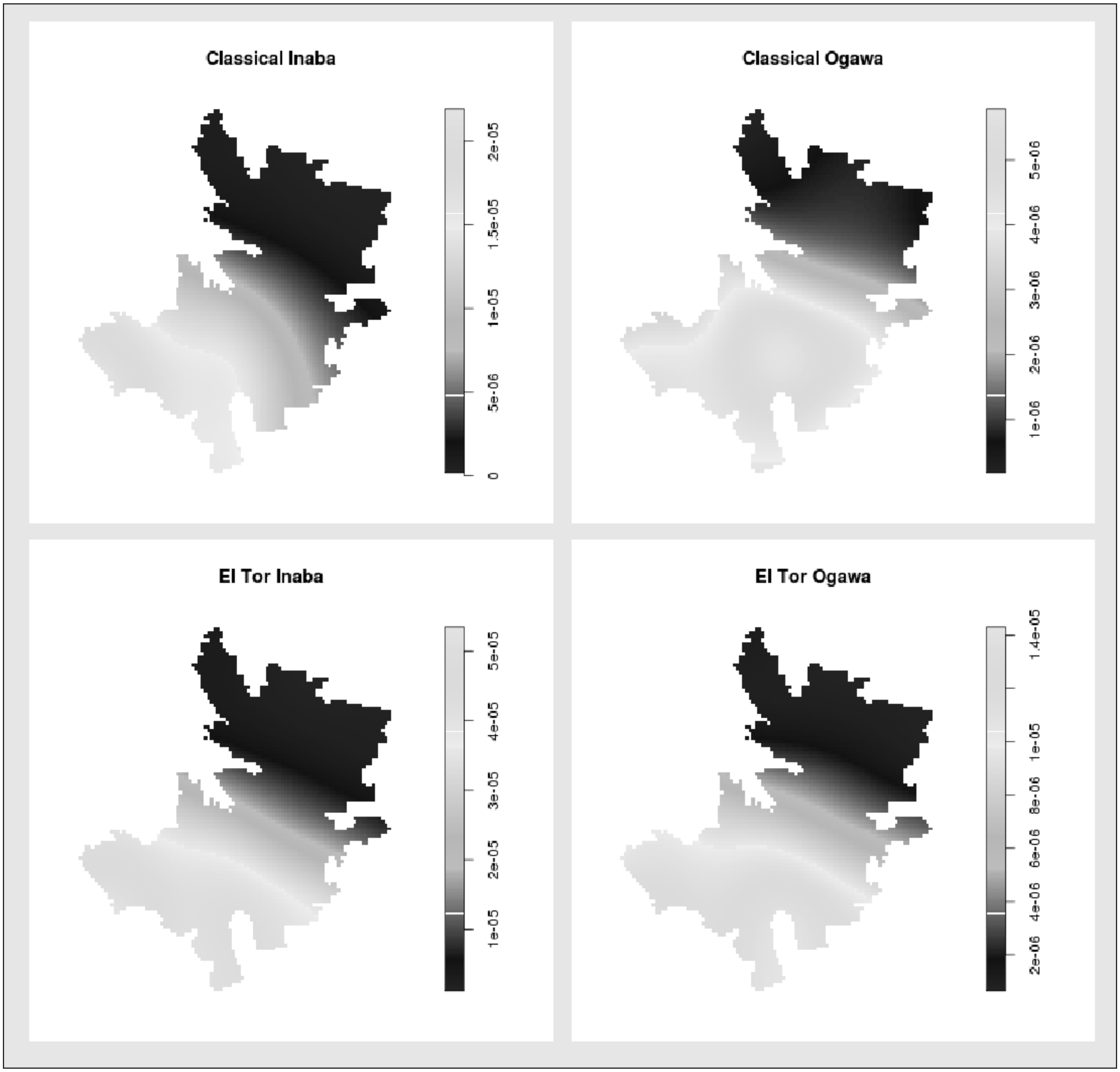

Figure S1: Spatial density of cholera cases.

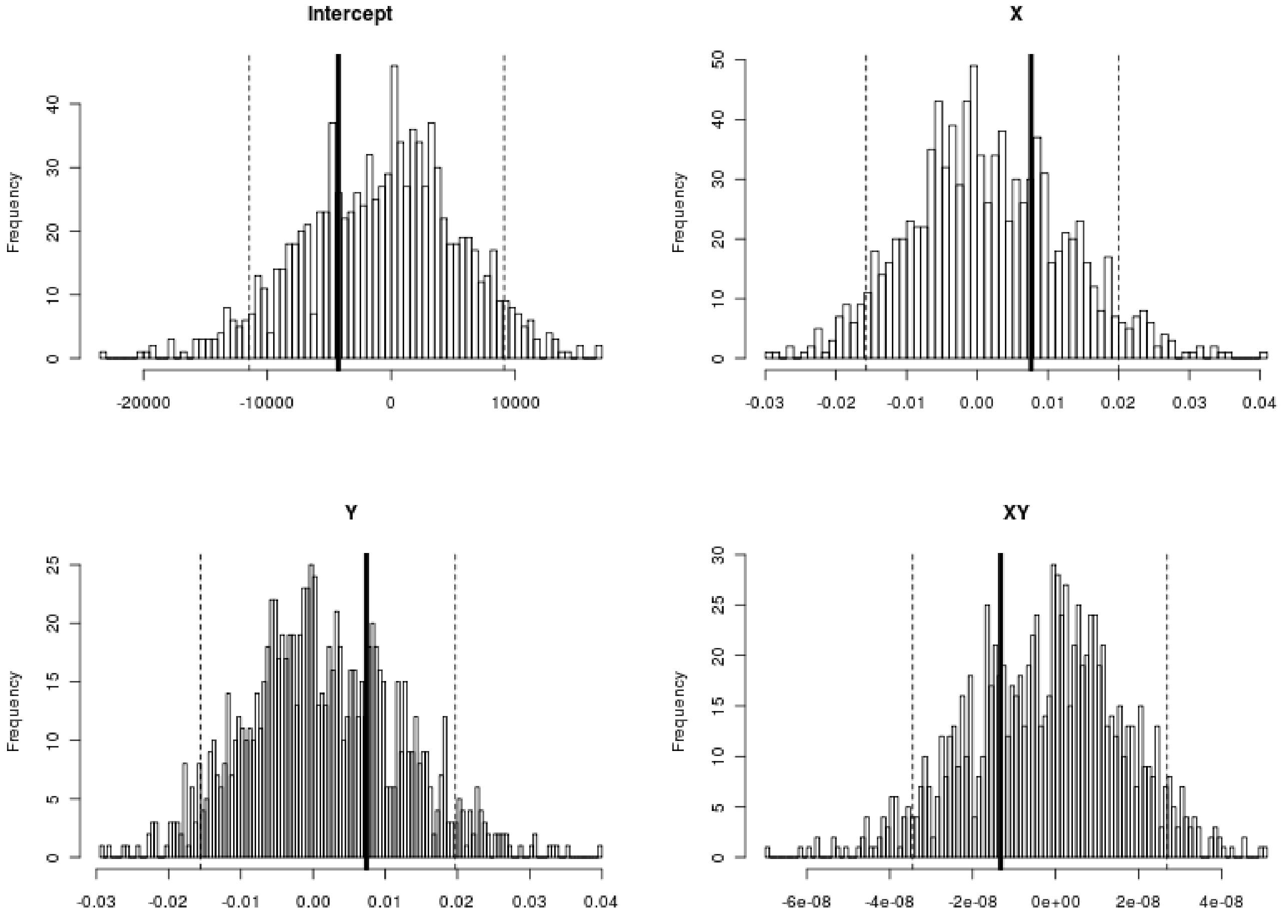

Figure S2: Spatial Trends for X, Y and XY directions for Classical Inaba cholera cases. Vertical black bold lines represent the observed trend values. White bars were obtained by 1000 Monte Carlo replications. Vertical dashed lines represent the 95% confidence interval these distributions.

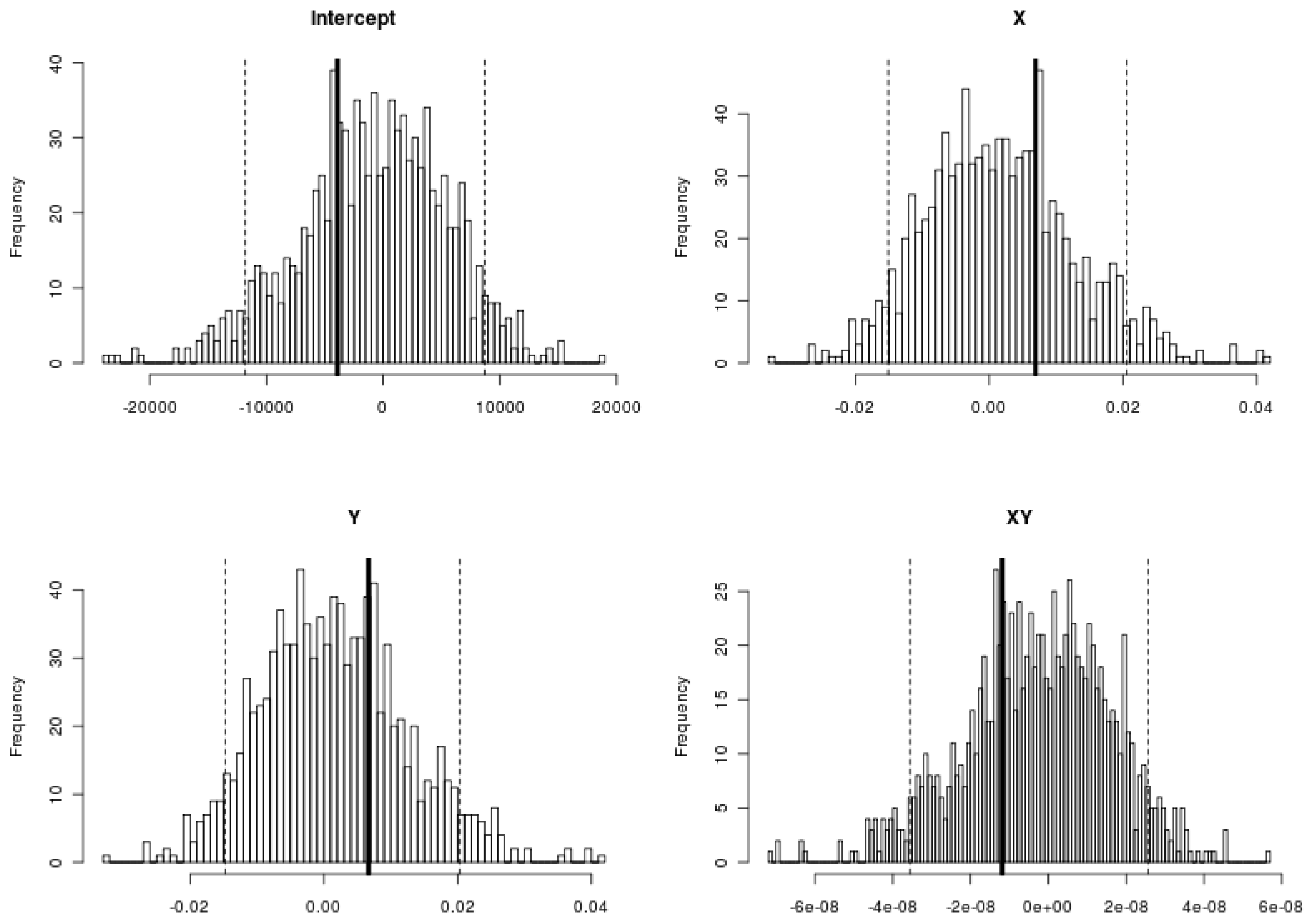

Figure S3: Spatial Trends for X, Y and XY directions for Classical Ogawa cholera cases. See caption of Figure S2 for details.

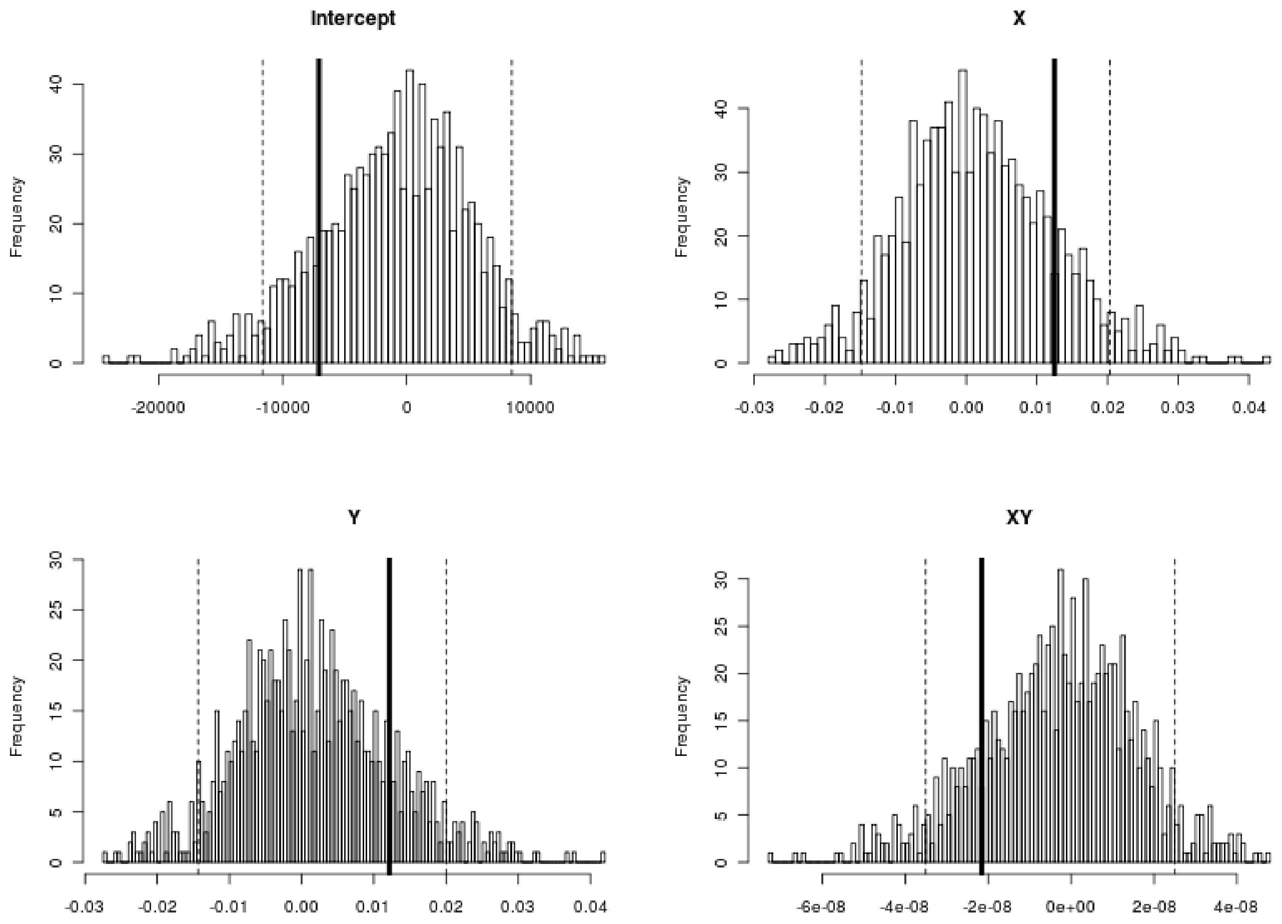

Figure S4: Spatial Trends for X, Y and XY directions for El Tor Inaba cholera cases. See caption of Figure S2 for details.

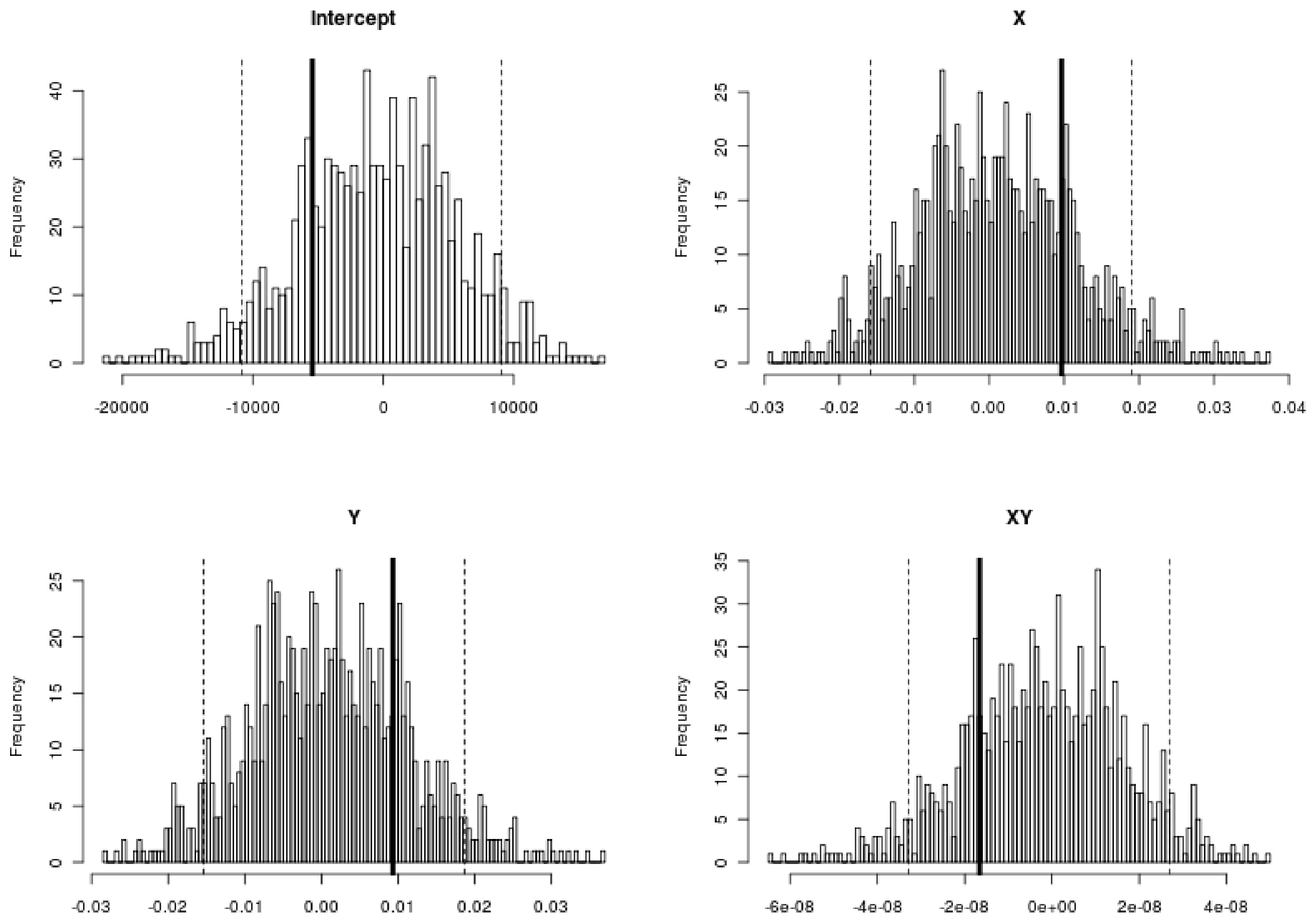

Figure S5: Spatial Trends for X, Y and XY directions for El Tor Ogawa cholera cases. See caption of Figure S2 for details.

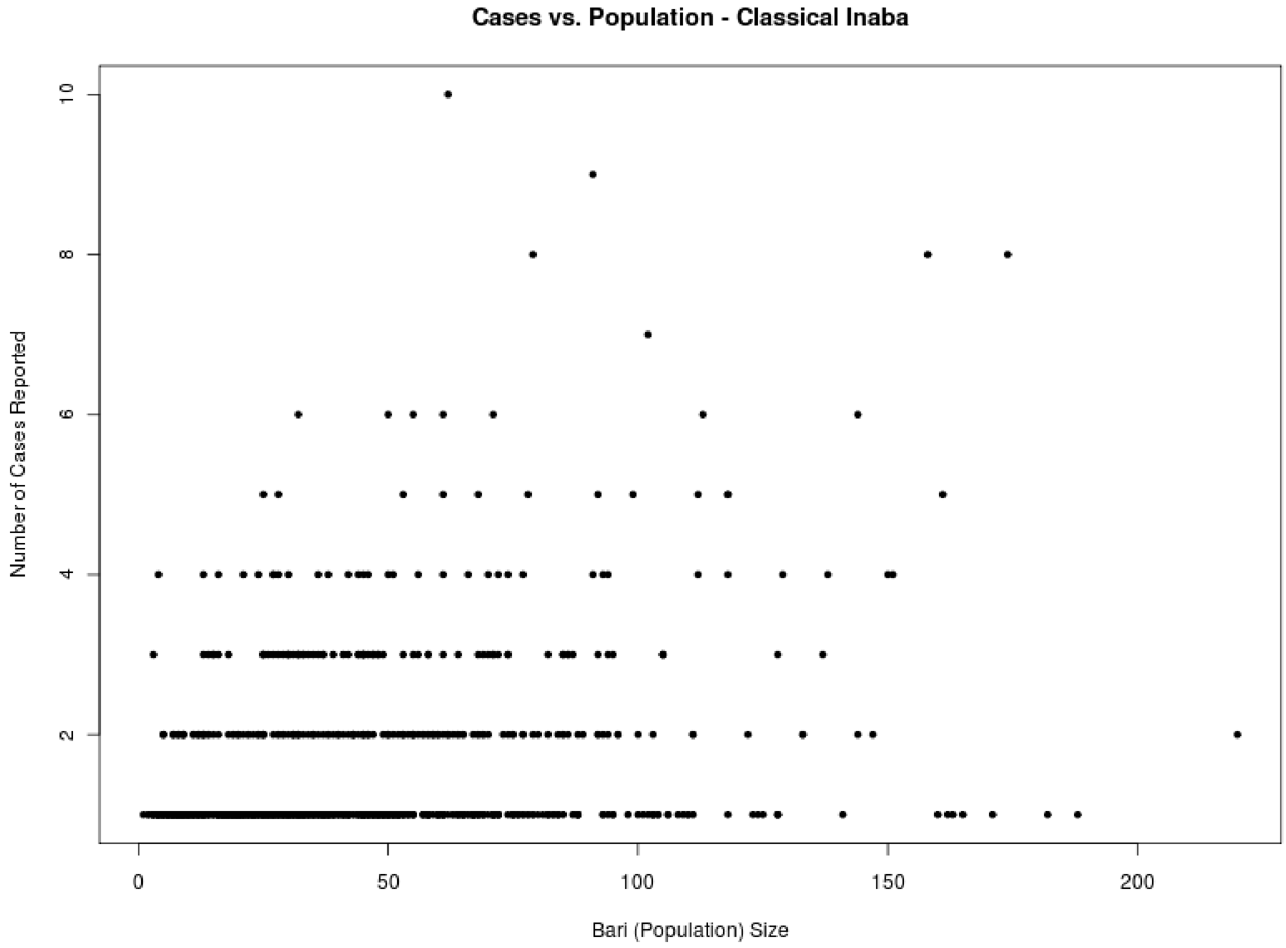

Figure S6: Number of Classical Inaba reported cases versus population size of the *baris*.

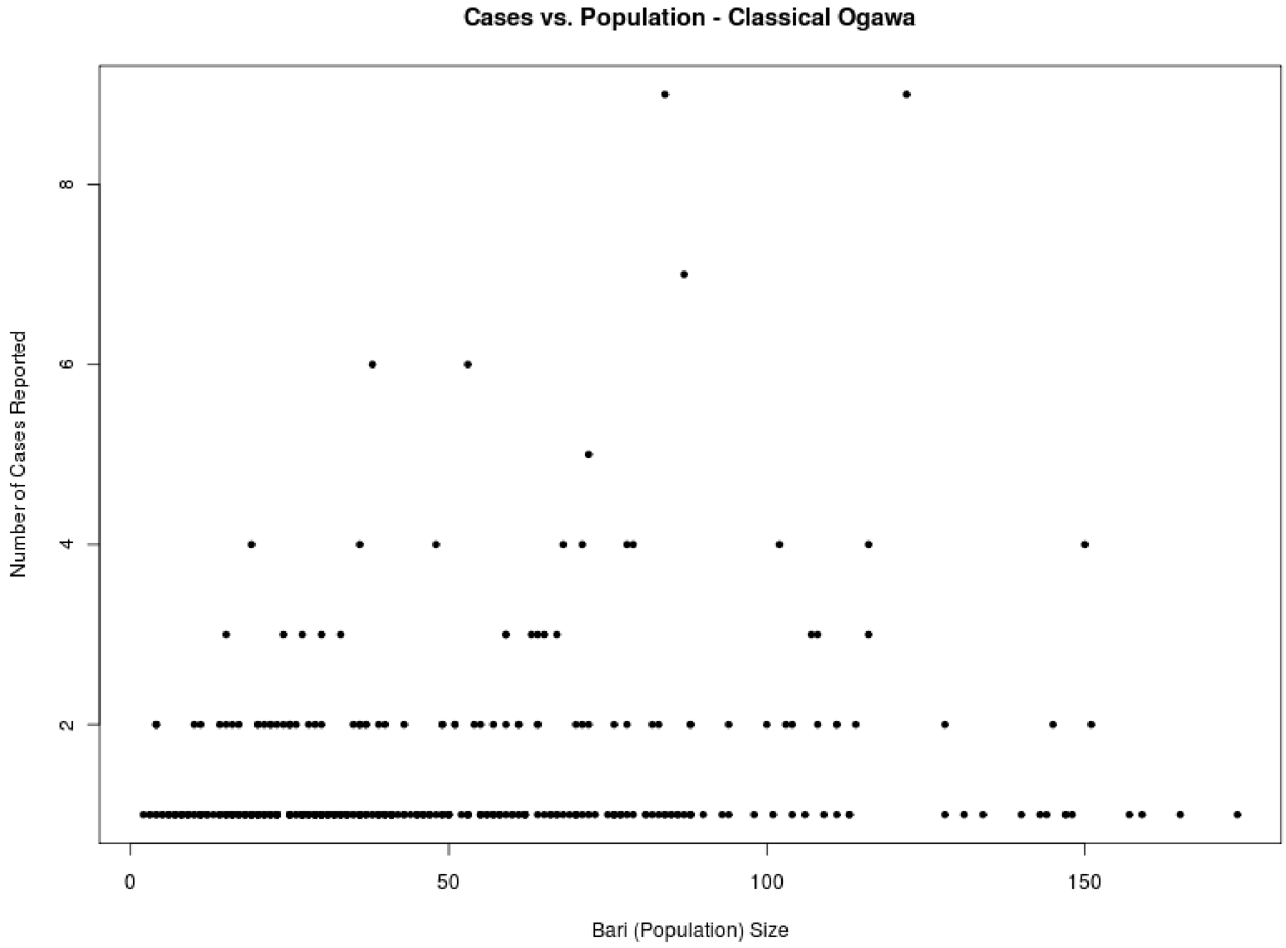

Figure S7: Number of Classical Ogawa reported cases versus population size of the *baris*.

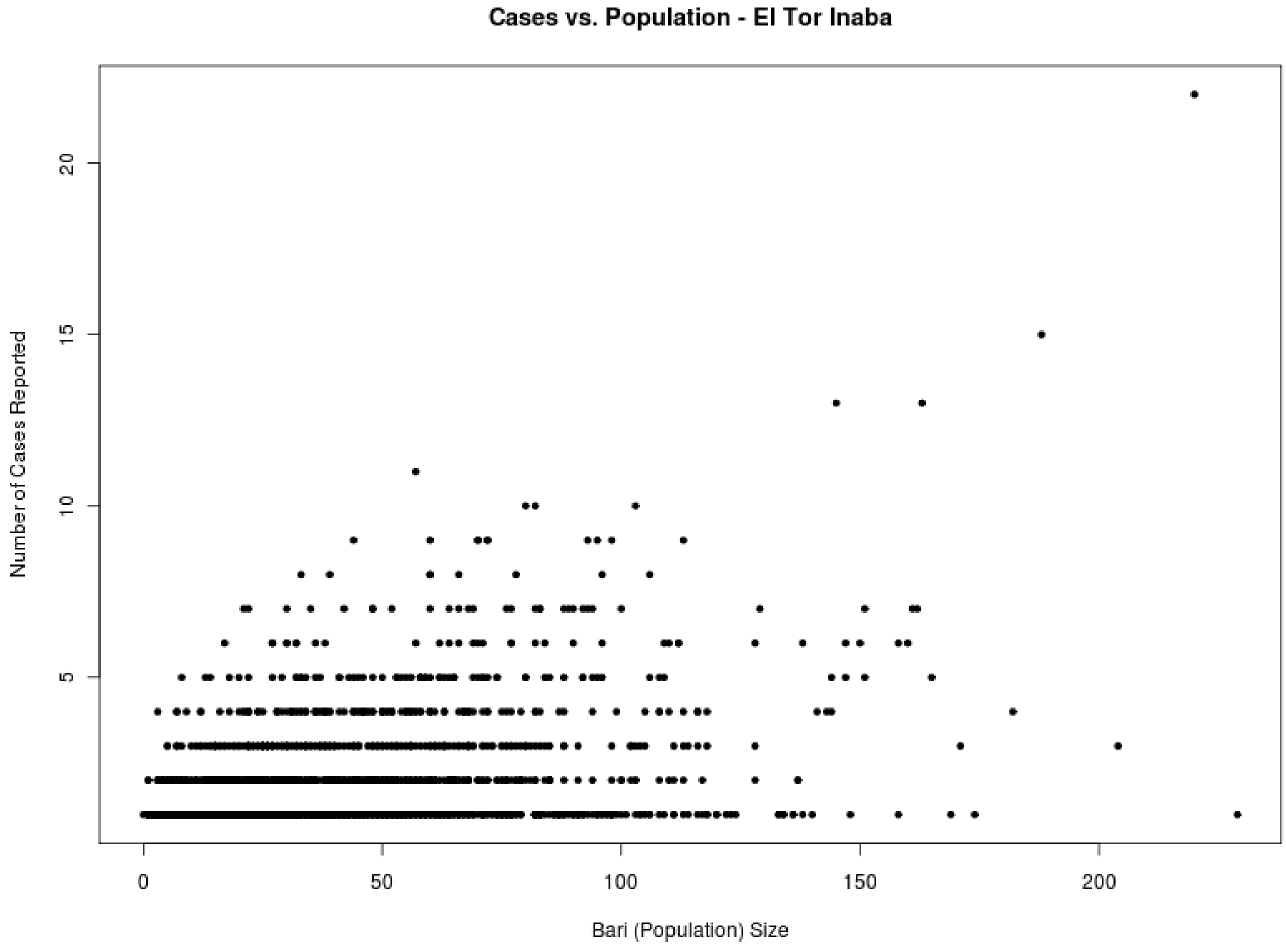

Figure S8: Number of El Tor Inaba reported cases versus population size of the *baris*.

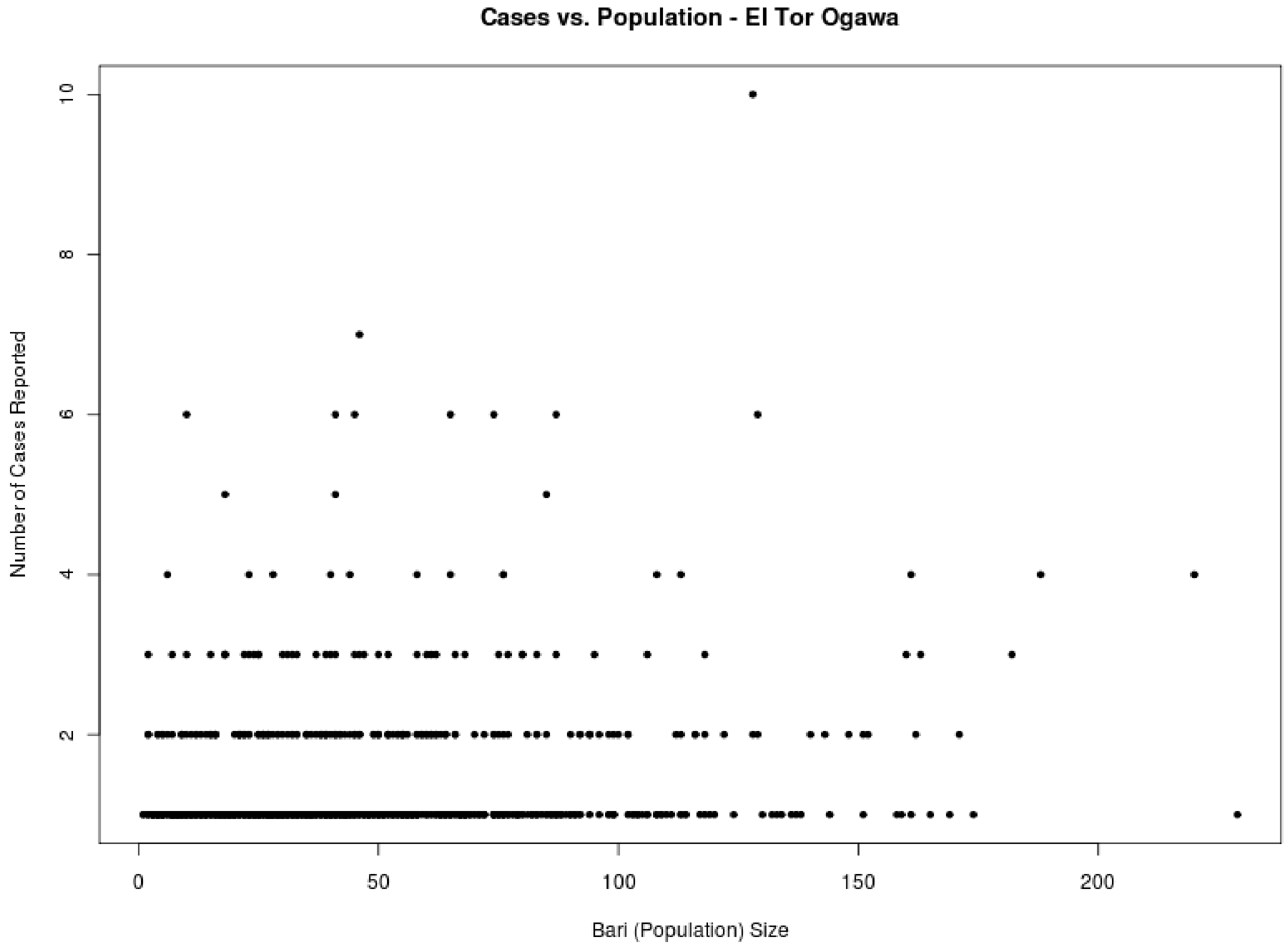

Figure S9: Number of El Tor Ogawa reported cases versus population size of the *baris*.
