# Supplementary material for: Spatial clustering in the spatio-temporal dynamics of endemic cholera
Source: BMC Infect Dis. 2010 Mar 6;10:51. doi: 10.1186/1471-2334-10-51 (PMC2846945; doi:10.1186/1471-2334-10-51)

Additional Figures Figure S10 - Clustering Size

Each Figure shows the clustering size of cases for different epidemics. Different symbols represent different temporal aggregation of the cases.

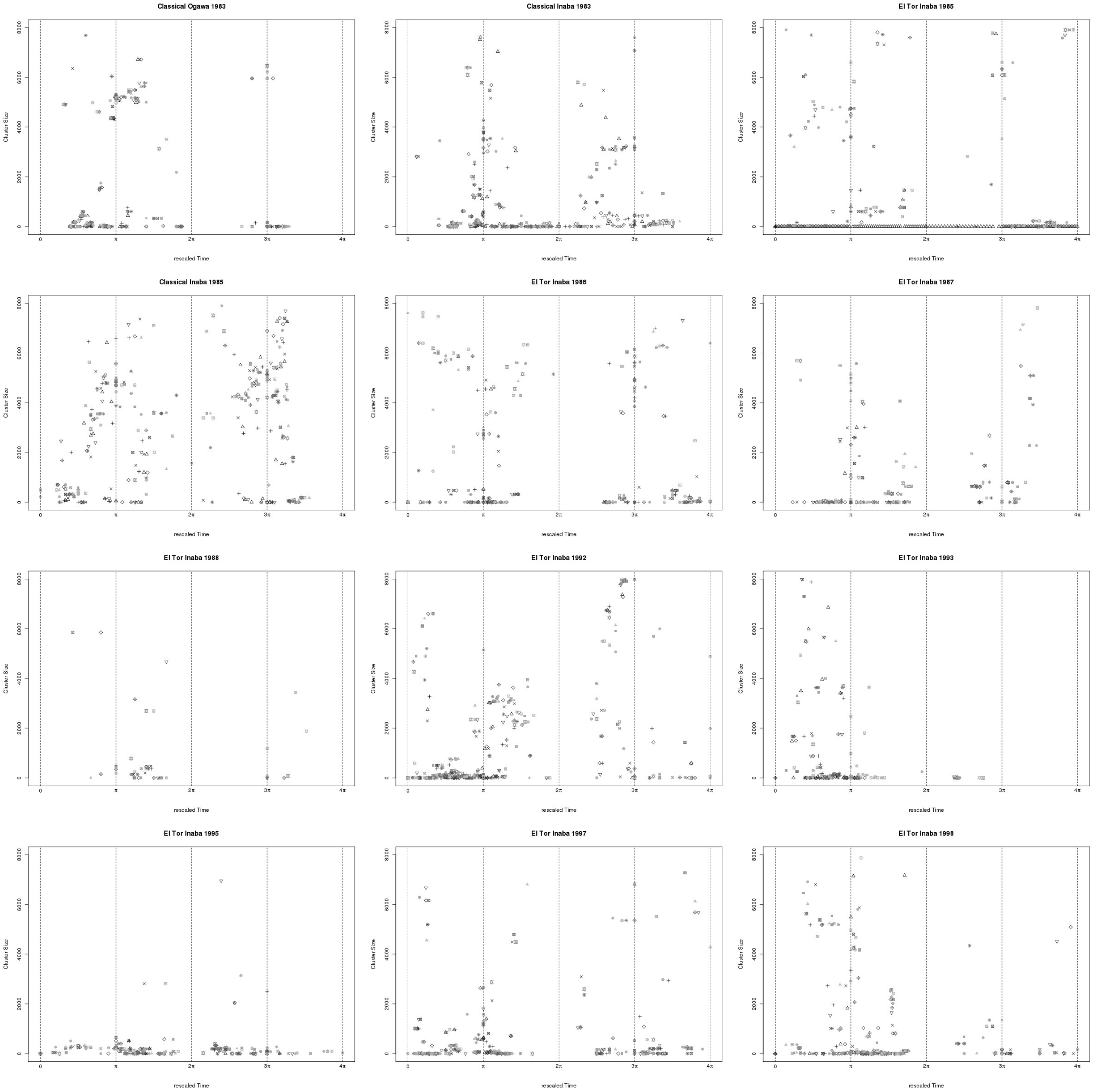

Supplement: Additional file 3 — Additional Figure S10, showing clustering size between cases. [file 1471-2334-10-51-S3.PDF]
