# Supplementary figures and images for: Spatial clustering in the spatio-temporal dynamics of endemic cholera
Source: BMC Infect Dis. 2010 Mar 6;10:51. doi: 10.1186/1471-2334-10-51 (PMC2846945; doi:10.1186/1471-2334-10-51)

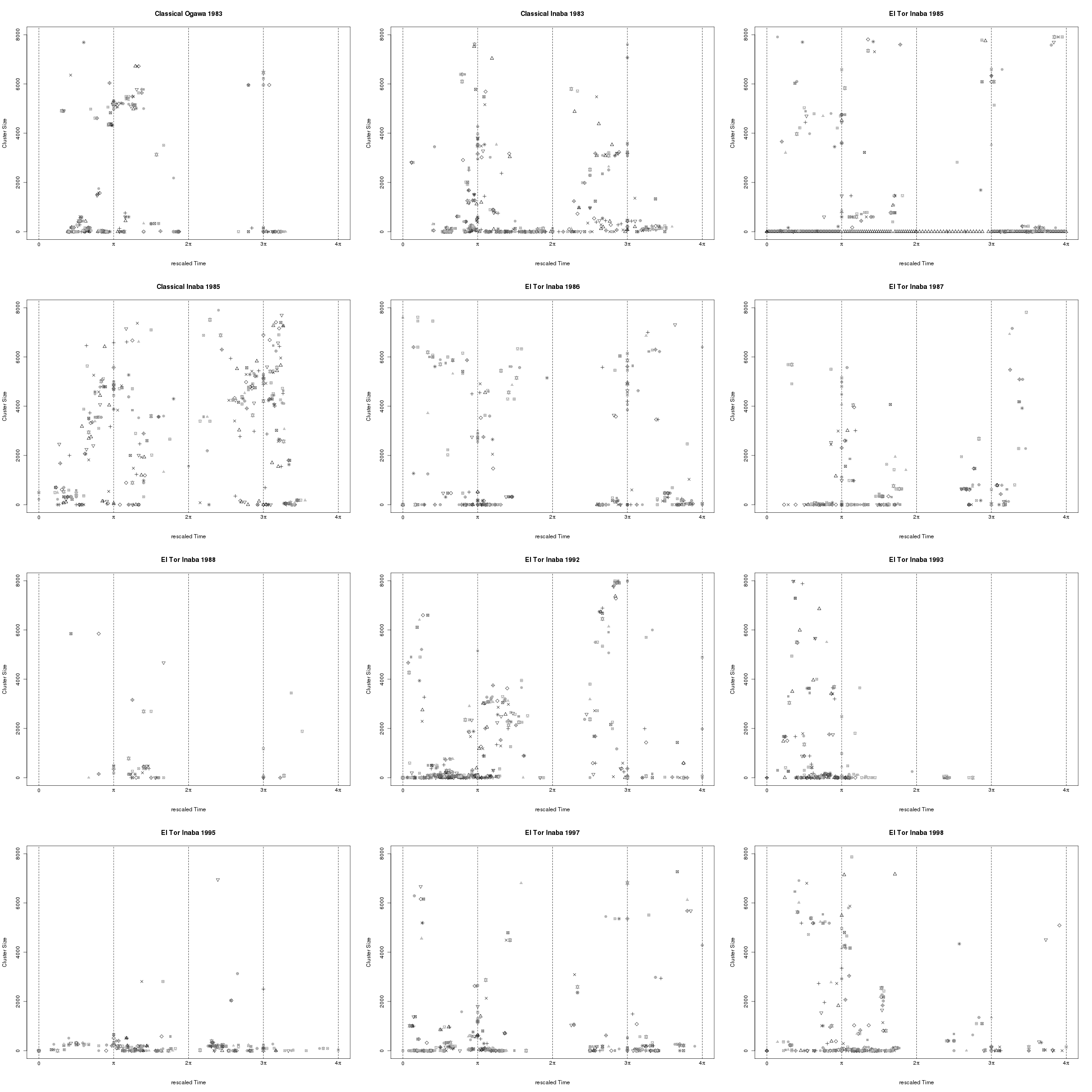

Supplement: Additional file 5 — High definition version of the addition Figure S11. [file 1471-2334-10-51-S5.PNG]

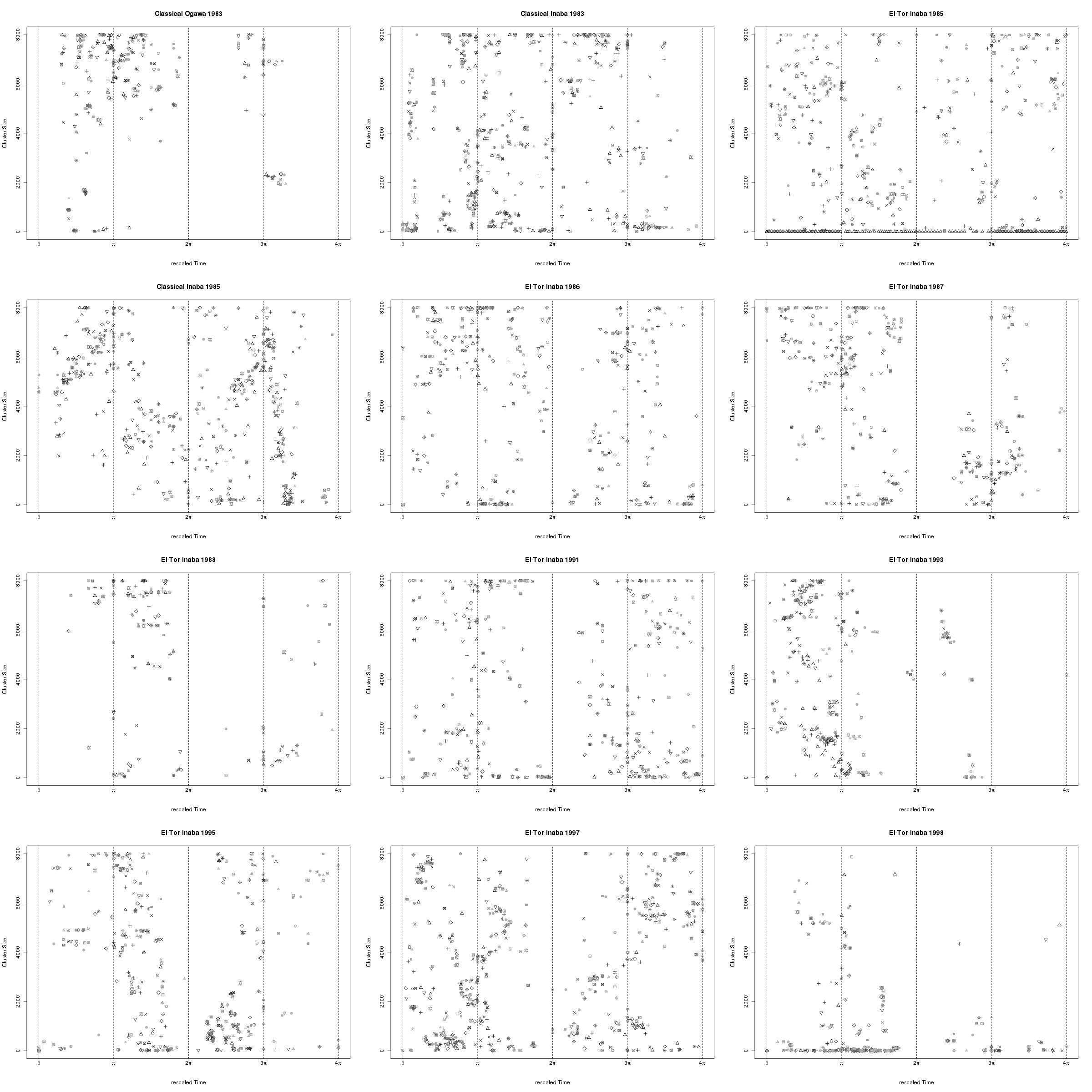

Supplement: Additional file 6 — High definition version of the addition Figure S10. [file 1471-2334-10-51-S6.PNG]
